# Morphologic Damage of Rat Alveolar Epithelial Type II Cells Induced by Bile Acids Could Be Ameliorated by Farnesoid X Receptor Inhibitor Z-Guggulsterone In Vitro

**DOI:** 10.1155/2016/9283204

**Published:** 2016-06-01

**Authors:** Jieqin Wang, Yaowei Huang, Xusheng Hou, Wenyu Wu, Lei Nie, Yinghong Tian, Yanmeng Lu, Yanru Yin

**Affiliations:** ^1^Department of Physiology, Southern Medical University, 1838 Guangzhou Avenue, North Baiyun District, Guangzhou, Guangdong 510080, China; ^2^Institute of Genetic Engineering, Southern Medical University, 1838 Guangzhou Avenue, North Baiyun District, Guangzhou, Guangdong 510080, China; ^3^Laboratory of Electron Microscopy, Southern Medical University, 1838 Guangzhou Avenue, North Baiyun District, Guangzhou, Guangdong 510080, China

## Abstract

*Objective*. To determine whether bile acids (BAs) affect respiratory functions through the farnesoid X receptor (FXR) expressed in the lungs and to explore the possible mechanisms of BAs-induced respiratory disorder.* Methods*. Primary cultured alveolar epithelial type II cells (AECIIs) of rat were treated with different concentrations of chenodeoxycholic acid (CDCA) in the presence or absence of FXR inhibitor Z-guggulsterone (GS). Then, expression of FXR in nuclei of AECIIs was assessed by immunofluorescence microscopy. And ultrastructural changes of the cells were observed under transmission electron microscope and analyzed by Image-Pro Plus software.* Results*. Morphologic damage of AECIIs was exhibited in high BAs group in vitro, with high-level expression of FXR, while FXR inhibitor GS could attenuate the cytotoxicity of BAs to AECIIs.* Conclusions*. FXR expression was related to the morphologic damage of AECIIs induced by BAs, thus influencing respiratory functions.

## 1. Introduction

Bile acids (BAs) were once considered to be just detergent molecules synthesized in the liver, which played an important role in the cholesterol homeostasis. However, recently, it has been discovered that BAs can also activate specific nuclear receptors and cell signaling pathways and function as nutrient signaling molecules [1]. Among these, the farnesoid X receptor (FXR) is one of the most extensively investigated agents. As a member of the “orphan” nuclear receptor superfamily, FXR is expressed mainly in the liver, intestine, kidney, and adrenal gland, with much lower levels in adipose tissue. It has been shown that FXR has a pivotal role in controlling bile acid homeostasis, lipoprotein and glucose metabolism, hepatic regeneration, intestinal bacterial growth, and the response to hepatotoxins [2].

Previous research suggested that BAs could interfere with the regulation of respiratory functions. Clinical data revealed that intrahepatic cholestasis of pregnancy (ICP) was always complicated by infant respiratory distress syndrome (IRDS) [3]. Afterwards, BAs were detectable in the bronchoalveolar lavage fluid (BALF) of these infants [4]. In addition, aspiration of duodenogastroesophageal refluxate is prevalent after lung transplantation and correlated with the development of bronchiolitis obliterans syndrome (BOS). Further, D'Ovidio et al. [5] demonstrated that elevated BALF BAs could promote early BOS development via an inflammatory process. Similarly, our earlier study showed that there was a significant change in the respiratory function in animals with high serum concentration of BAs. BAs in the serum can have direct effects on respiratory functions besides reflex responses [6]. Since BAs are the endogenous ligand of FXR, we speculate that BAs may regulate respiratory functions by activating the nuclear receptor. In in vitro experiment, we confirmed that GW4064 and four bile acids chenodeoxycholic acid (CDCA), lithocholic acid (LCA), cholic acid (CA), and deoxycholic acid (DCA) except ursodeoxycholic acid (UDCA) all exerted significant effects on the spontaneous periodic respiratory-related rhythmical discharge activity (RRDA) recorded from hypoglossal nerves in a concentration-dependent manner. However, such effects could be reversed by FXR inhibitor Z-guggulsterone (GS) [7]. These evidences strongly support our original hypothesis. Taking into account the extensive effect of BAs, we further assume that BAs can also affect respiratory functions through FXR expressed in the lungs besides the respiratory center.

Pulmonary surfactant is a mixture of lipids and proteins, which is synthesised by alveolar epithelial type II cells (AECIIs) and stored in lamellar bodies (LBs). It can lower surface tension, thereby reducing the work of breathing and preventing alveolar collapse. Moreover, pulmonary surfactant plays a major role in innate immune defence [8]. The presence of BAs in the alveoli can influence the hydrolysation of phosphatidylcholines catalyzed by phospholipase A2 (PLA2) and lower surfactant protein A (SP-A) and D (SP-D) concentrations, leading to surfactant dysfunction and lung tissue inflammation [9–11]. Previous studies also confirmed that BAs can inhibit surfactant secretion [12–15]. In this research, we observed and analyzed the ultrastructural changes of AECIIs after treatment of CDCA in the absence or presence of the FXR blocker GS to investigate the possible mechanisms of BAs-induced lung disorder.

## 2. Materials and Methods

### 2.1. Ethics Statement and Animals

Adult male Sprague-Dawley rats (180–200 g) were obtained from the Laboratory Animal Center of Southern Medical University (SCXK 2011-0015). All the experimental procedures were in compliance with the National Institutes of Health Guidelines for Care and Use of Laboratory Animals and received approval from the Bioethics Committee of Southern Medical University.

### 2.2. Reagents

Trypsin was purchased from Solarbio (Beijing, China), DNase I was purchased from Roche Diagnostics GmbH (Mannheim, Germany), DMEM/F-12 was purchased from Thermo Fisher Scientific Inc. (Waltham, MA, USA), newborn calf serum was purchased from Shanghai ExCell Biology, Inc. (Shanghai, China), dimethyl sulfoxide was purchased from Biosharp (Hefei, China), chenodeoxycholic acid (CDCA) was purchased from Shanghai Yiji Co., Ltd. (Shanghai, China), Z-guggulsterone, rabbit polyclonal anti-FXR antibody, and DAPI were purchased from Santa Cruz Biotechnology (Santa Cruz, CA, USA), and rat IgG and DyLight® 488-conjugated goat anti-rabbit IgG were purchased from EarthOx, LLC (Millbrae, California, USA).

### 2.3. Isolation of AECIIs

The method of cell isolation was a modification of the method developed by Dobbs et al. [16, 17]. Adult male Sprague-Dawley rats (180–200 g) were anesthetized with pentobarbital (50 mg/kg/body weight) and heparin (400 U/kg/body weight). Peritoneal cavity was exposed, and the diaphragm was incised to deflate the lungs, followed by transecting the inferior vena cava and aorta. The chest was then opened and the thyroid gland was removed. After cannulating the trachea, the left atrium was nicked, and the pulmonary artery was cannulated and perfused with solution 2 (140 mM NaCl, 5 mM KCl, 2.5 mM Na_2_HPO_4_·12H_2_O, 10 mM hepes, 6 mM glucose, 0.2 mM EDTA, 1.3 mM MgSO_4_·7H_2_O, and 2 mM CaCl_2_; pH 7.4). The lungs, as well as the heart, were carefully dissected away from the chest cavity, and the tracheal cannula was used to lavage twice with solution 1 (140 mM NaCl, 5 mM KCl, 2.5 mM Na_2_HPO_4_·12H_2_O, 10 mM hepes, 6 mM glucose, and 0.2 mM EDTA; pH 7.4). Subsequently, the lungs were digested by instilling 0.1% trysin solution (trysin was dissolved in solution 1) containing 20 *μ*g/mL DNase I in a 37°C water bath and incubating for 30 min. Then the trachea and heart were removed, and the lung parenchyma was minced quickly with an ophthalmologic scissor and 50 *μ*L 10 *μ*g/*μ*L DNase I was added to minimize cell clumping. Afterwards, the lung pieces were suspended in DMEM/F-12 with 10% newborn calf serum, and the proteolytic suspension was filtered through 74 *μ*m nylon mesh and centrifuged to collect dissociated cells. The dissociated cells were then resuspended in DMEM/F-12 with 10% newborn calf serum and incubated (37°C and 5% CO_2_) in culture flasks coated with rat IgG for 1 hr. Thereafter, nonadherent cells consisting primarily of AECIIs were collected and washed with DMEM/F-12 three times. Finally, the cells were resuspended in DMEM/F-12 with 10% newborn calf serum and cultured for an additional 24 hr at a concentration of 1 × 10^6^ cells/mL before further experiments. Cell viability was determined by exclusion of trypan blue dye, and purity was evaluated by tannic acid staining under light microscope [18]. The purity and viability of AECIIs from our isolation procedure were consistently greater than 92%.

### 2.4. Experimental Protocol

Harvested cells were divided into 12 groups: Group 1 was treated with dimethyl sulfoxide (DMSO, final concentration was 0.2%) for 6 hr as control. Groups 2–4 were treated with 25, 100, and 200 *μ*M CDCA, respectively, for 6 hr to observe the effects of different concentrations of CDCA on AECIIs. Group 5 was treated with 40 *μ*M GS for 6 hr to observe the effects of FXR blocker GS on AECIIs. Groups 6–8 were treated with the above three concentrations of CDCA together with 40 *μ*M GS for 6 hr to observe the effects of combination of CDCA and GS on AECIIs. In order to exclude the possibility that GS could interact with CDCA which prevented the latter from finally binding to FXR, Groups 9–12 were treated with GS for 6 hr before later addition of different concentrations of CDCA. All the drugs were dissolved in DMSO.

### 2.5. Immunocytochemistry Analysis of FXR Expression

Immunofluorescence staining was used to demonstrate the presence of FXR in nuclei of AECIIs and assess the expression of the nuclear receptor. The cells were fixed in 4% paraformaldehyde and permeabilized in 0.25% Trinton X-100, followed by incubation with rabbit polyclonal anti-FXR antibody (1 : 50 dilution) and DyLight 488-conjugated goat anti-rabbit IgG (1 : 200 dilution). To visualize the nuclei, the cells were counterstained with DAPI (0.5 mg/mL). Fluorescence was observed in a Nikon Eclipse 80i microscope.

### 2.6. Ultrastructural Observation of AECIIs

The cells were prefixed in 2.5% glutaraldehyde overnight, followed by a postfixation with 1% osmium tetroxide for 1-2 hr. The specimens were dehydrated in gradient acetone, incubated in propylene oxide/epoxy resin mixture, and embedded in pure resin. Afterwards, 60–90 nm sections were cut and double stained with uranyl acetate and lead citrate and then observed under Hitachi H-7500 transmission electron microscope. AECIIs were identified by their short microvillus and LBs. And ten photos were taken for each ultrathin section. Characteristics of the cells were observed at high power field. Image-Pro Plus 7.0 was used for measurement.

### 2.7. Statistical Analysis

SPSS 20.0 software was adopted to proceed with statistical analysis, and the results were presented as sample mean ± standard deviation. One-way ANOVA was used to assess multiple groups of data and LSD *t*-test was used to compare between groups of multiple groups. *P* < 0.05 indicates a significant difference.

## 3. Results

### 3.1. Immunofluorescence Analysis of FXR Expression

**Figure 1:**
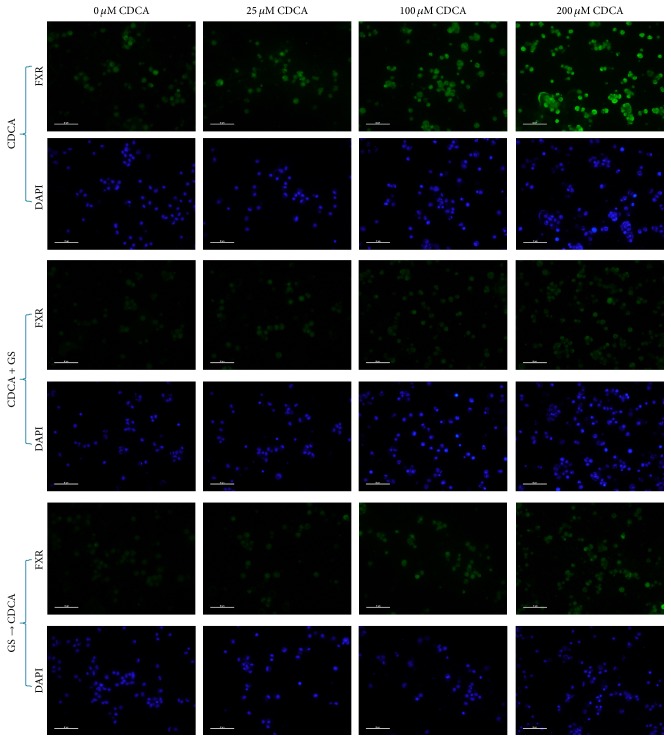
Representative micrographs of FXR immunostaining of CDCA-treated primary cultured AECIIs. Rabbit polyclonal anti-FXR antibody demonstrated the presence of FXR in nuclei of AECIIs. And the fluorescence intensity on AECIIs treated with GS was much lower than that without GS. Magnification bar: 50 *μ*m.

FXR was demonstrated in nuclei of AECIIs by immunofluorescence microscopy with a rabbit polyclonal anti-FXR antibody. And not unexpectedly, as the most potent natural activator of FXR [19], CDCA caused a concentration-dependent increase in the fluorescence intensity on AECIIs. However, the intensity of fluorescence declined markedly after treatment of CDCA in the presence of GS. Same results were observed after exposure to GS first following addition of CDCA (Figure 1).

### 3.2. Ultrastructural Observation

**Figure 2:**
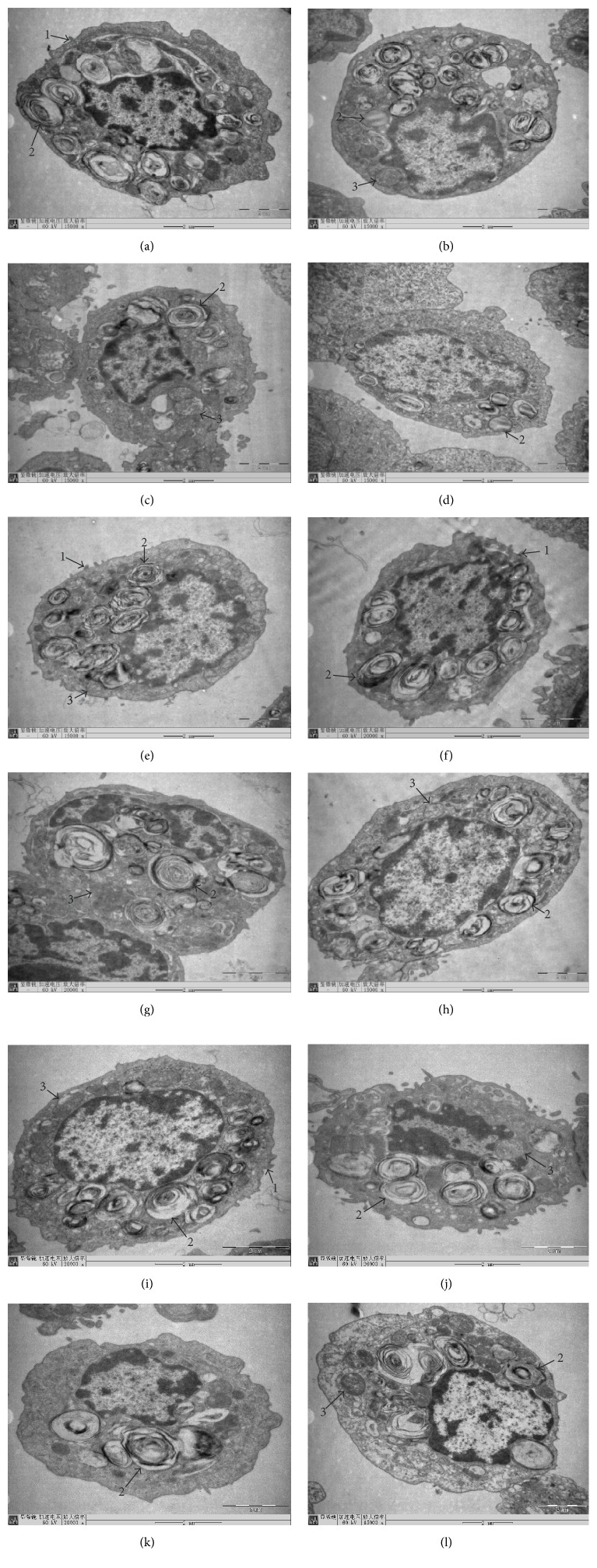
The morphology of primary cultured AECIIs of rat after treatment of CDCA and the FXR blocker GS under transmission electron microscope. In Group 1 (control), Group 5 (40 *μ*M GS, MIX), and Group 9 (40 *μ*M GS, PRE), AECIIs were in good condition. Large numbers of LBs were observed, with dark color and ring-like arrangement (×15000, ×15000, and ×20000, (a), (e), and (i)). In Group 2 (25 *μ*M CDCA), mitochondria had slight swelling, with some LBs demonstrating vacuolization (×15000, (b)). In Group 3 (100 *μ*M CDCA), the number of LBs reduced significantly. Mitochondria swelled seriously and showed balloon-like change (×15000, (c)). In Group 4 (200 *μ*M CDCA), fewer LBs were presented, showing vacuole-like deformity. Microvilli on the surface had disappeared (×15000, (d)). In Groups 6–8 and Groups 10–12 (25, 100, and 200 *μ*M CDCA with 40 *μ*M GS, MIX, and PRE), the number of LBs markedly increased compared to Groups 2–4, with clear lamellar and cellular structure (×20000, ×20000, ×15000, ×20000, ×20000, and ×15000, (f), (g), (h), (j), (k), and (l)). Arrow 1: microvillus; arrow 2: lamellar body; arrow 3: mitochondria.

In Group 1 (control), Group 5 (40 *μ*M GS, MIX), and Group 9 (40 *μ*M GS, PRE), AECIIs had clear structure, with oval appearance and short microvillus on its surface. The karyolemma was complete, and the chromatin inside the nucleus was homogeneous. Abundant LBs could be observed, with uniform density and ring-like arrangement (Figures 2(a), 2(e), and 2(i)). In Group 2 (25 *μ*M CDCA), normal structure of AECIIs was seen, still with substantial LBs, though some demonstrated vacuolization. Mitochondria had slight swelling and enlarged volume (Figure 2(b)). In Group 3 (100 *μ*M CDCA), AECIIs were round, and the number of LBs reduced significantly. Mitochondria swelled seriously, some showed balloon-like change, and crista cavitation vanished (Figure 2(c)). In Group 4 (200 *μ*M CDCA), the number of LBs was further reduced, lots of which exhibited vacuole-like deformity, with disappearance of microvillus structure on cell surface (Figure 2(d)). In Groups 6–8 and Groups 10–12 (25, 100, and 200 *μ*M CDCA with 40 *μ*M GS, MIX, and PRE), the number of LBs markedly increased compared to Groups 2–4. Clear lamellar and cellular structure was displayed (Figures 2(f), 2(g), 2(h), 2(j), 2(k), and 2(l)).

### 3.3. Comparison of the Sectional Area Ratio of LBs to Cytoplasm of AECIIs 

**Figure 3:**
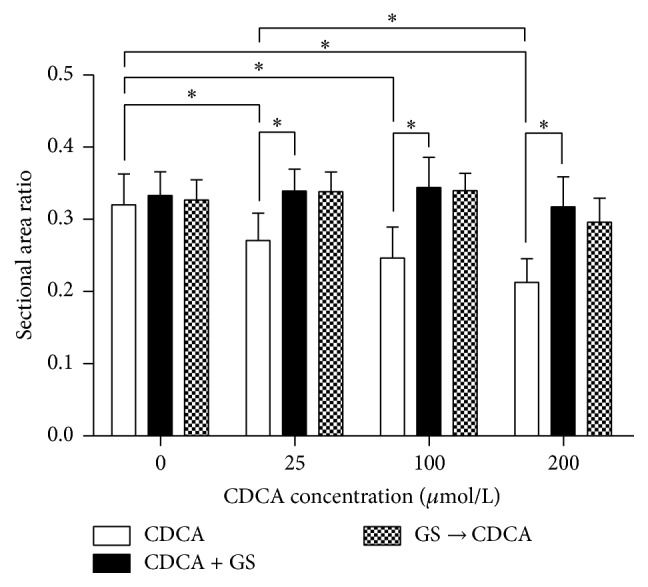
Effects of different concentrations of CDCA on the sectional area ratio of LBs to cytoplasm of primary cultured AECIIs in the absence or presence of the FXR blocker GS. CDCA was found to dose-dependently reduce the sectional area ratio of LBs to cytoplasm of AECIIs. However, such effects were entirely reversed by FXR inhibitor GS. ^*∗*^ *P* < 0.01.

Our results showed that CDCA produced a dose-dependent decrease in the sectional area ratio of LBs to cytoplasm of rat AECIIs in vitro. Compared to the control, the sectional area ratio of LBs to cytoplasm of AECIIs was significantly decreased by 15.52%, 23.00%, and 33.61% separately at the concentrations of 25 *μ*M, 100 *μ*M, and 200 *μ*M; *P* < 0.01. However, after exposure to the above three concentrations of CDCA together with 40 *μ*M GS, the sectional area ratio of LBs to cytoplasm of AECIIs was markedly increased by 25.52%, 39.62%, and 49.24% separately compared to the same concentration of CDCA without GS; *P* < 0.01. Meanwhile, no statistical differences among Group 1, Groups 5–8, and Groups 9–12 were observed; *P* > 0.05. It meant that the reduction in the sectional area ratio of LBs to cytoplasm of AECIIs caused by CDCA could be completely reversed by FXR inhibitor GS (Figure 3).

## 4. Discussion

**Figure 4:**
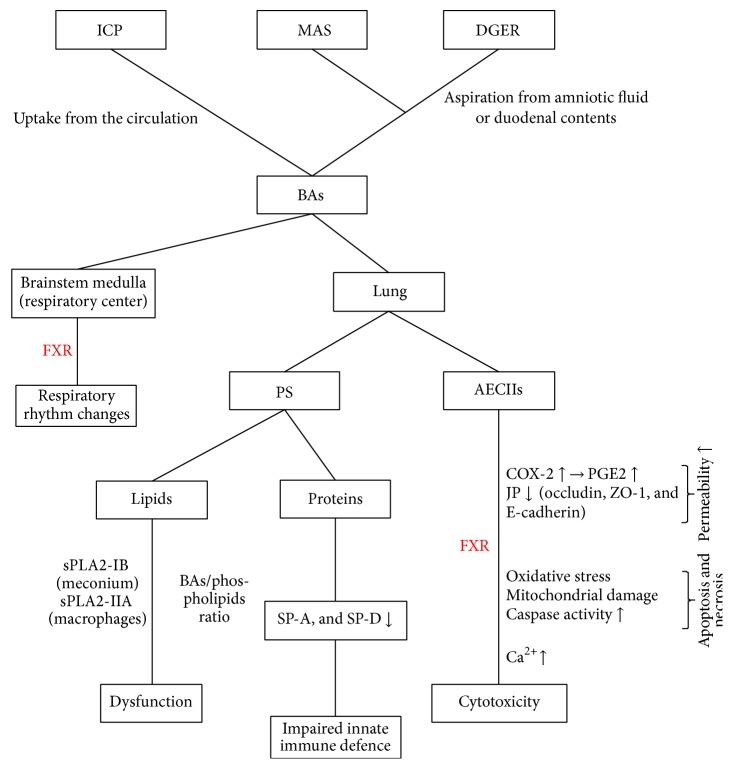
Possible mechanisms of BAs-induced respiratory disorder.

As mentioned previously, effects of BAs on the respiratory function have received increasing attention in recent years. BAs can reach the lungs in two distinct pathways: uptake from the circulation during ICP [20] and aspiration from amniotic fluid during MAS [21] or duodenal contents during duodenogastroesophageal reflux (DGER) [5]. However, a retrospective study revealed that higher maternal bile acid levels were correlated significantly with meconium-stained amniotic fluid [22]. In other words, such two pathways are not completely independent. Zecca et al. [11] once proposed three theories of BAs-induced lung injury. First, BAs may cause surfactant alteration by affecting the hydrolysation of phosphatidylcholines catalyzed by PLA2. Second, a direct chemical damage of the lung epithelium produced by BAs may lead to the injury of enzymatic activities in AECIIs and increase the cellular cationic permeability. Last, BAs can lower SP-A and SP-D concentrations, contributing to impaired lung immunity and local inflammation. Actually, we believe it is the result of numerous varieties of complex mechanisms (Figure 4).

Our previous research [7] indicated that apart from UDCA, CDCA, DCA, LCA, and CA all exerted effects on RRDA recorded from hypoglossal nerves in a concentration-dependent manner. Respiratory cycle (RC), inspiratory time (IT), expiratory time (ET), and integral amplitude (IA) were influenced and such effects could be reversed by GS. These suggest that BAs may regulate respiratory functions through FXR located in the respiratory center. In this experiment, we first demonstrated the presence of FXR in nuclei of AECIIs by immunofluorescence microscopy. Not surprisingly, high CDCA group incorporated more FXR than the low one, and GS was proved to suppress FXR expression induced by CDCA. Then we observed and analyzed the ultrastructural changes of the cells under transmission electron microscope. Consequently, CDCA was found to damage the morphology of rat AECIIs in vitro in a concentration-dependent manner. In high dose groups, the number of LBs reduced significantly, lots of which demonstrated vacuolization, with disappearance of microvillus structure on cell surface. Mitochondria swelled seriously, some showed balloon-like change, and crista cavitation vanished. Moreover, CDCA produced a dose-dependent decrease in the sectional area ratio of LBs to cytoplasm of AECIIs. These results appeared to be similar to those reported earlier by Yu et al. [14] who studied the effects of BAs on fetal lung in rat model of ICP. Interestingly, we found that FXR inhibitor could influence damage to the morphology of AECIIs caused by BAs. After treatment of the mixture of CDCA and GS, the number of LBs markedly increased and the reduction in the sectional area ratio of LBs to cytoplasm was completely reversed, with clear lamellar and cellular structure. Same outcomes were exhibited after sufficient exposure to GS before later addition of CDCA, which ruled out the possibility that GS could form a sort of complex with the BAs so that the latter was unavailable to FXR.

We speculate that it is the cytotoxicity of BAs that accounts for the morphologic damage of AECIIs shown in our experiment. Several researches have confirmed the cytotoxicity of BAs to AECIIs (Figure 4). Zhangxue et al. [15] reported that glycochenodeoxycholate (GCDC) could induce AECIIs death via oxidative stress, mitochondrial damage, increased caspase activity, and inhibited surfactant secretion, while antioxidants, apoptosis, and necrosis inhibitors could rescue AECIIs death from GCDC stimulation. Su et al. [13] demonstrated that BAs maybe produced a dose-dependent increase in alveolar epithelial permeability, which was correlative with upregulation of MAPK, cPLA2, COX-2, and PGE2 generation and decay of occludin, ZO-1, and E-cadherin. Oelberg et al. [12] proved that bile salts at concentrations less than the critical micellar concentration (CMC) would produce cytotoxicity in association with Ca^2+^ accumulation in AECIIs, which was possibly responsible for the pathogenesis of meconium aspiration pneumonitis.

Since FXR expression was closely correlated with the ultrastructural changes of AECIIs, we presume that activation of FXR may be in part responsible for the cytotoxicity of BAs to AECIIs. And pulmonary surfactant is probably involved in this process. FXR activation by BAs may result in inhibited surfactant secretion, which is characterized by damage to the ultrastructure of LBs as well as by reduction in the number of LBs and the sectional area ratio of LBs to cytoplasm. Pulmonary surfactant is an essential lipid-protein complex for maintaining an optimal respiratory surface at the lungs, which is synthesised by AECIIs and stored in LBs [8]. Deficiency of pulmonary surfactant is prone to develop IRDS [23]. Therefore, FXR may induce surfactant inactivation, thus contributing to respiratory diseases.

In summary, we confirm the cytotoxicity of BAs to AECIIs under transmission electron microscope and firstly ascertain that BAs may affect respiratory functions by activating FXR expressed in the lungs. Besides, surfactant inactivation mediated by FXR is probably implicated in this process. These suggest that FXR inhibitor has great potential as a future therapeutic strategy against BAs-induced lung injury. However, due to our limitations, no exact pathways are given. There are few reports about the effect of FXR on respiratory functions. Thus, future studies are warranted to clarify the underlying mechanisms involved.

## References

[1] Hylemon P. B., Zhou H., Pandak W. M., Ren S., Gil G., Dent P. (2009). Bile acids as regulatory molecules. *Journal of Lipid Research*.

[2] Lee F. Y., Lee H., Hubbert M. L., Edwards P. A., Zhang Y. (2006). FXR, a multipurpose nuclear receptor. *Trends in Biochemical Sciences*.

[3] Zecca E., De Luca D., Marras M., Caruso A., Bernardini T., Romagnoli C. (2006). Intrahepatic cholestasis of pregnancy and neonatal respiratory distress syndrome. *Pediatrics*.

[4] Zecca E., De Luca D., Baroni S., Vento G., Tiberi E., Romagnoli C. (2008). Bile acid-induced lung injury in newborn infants: a bronchoalveolar lavage fluid study. *Pediatrics*.

[5] D'Ovidio F., Mura M., Tsang M. (2005). Bile acid aspiration and the development of bronchiolitis obliterans after lung transplantation. *Journal of Thoracic and Cardiovascular Surgery*.

[6] Wang F., Zhao C., Tian Y., Yin Y. (2013). Effect of high blood levels of bile acid on respiratory functions of New Zealand rabbits. *Nan Fang Yi Ke Da Xue Xue Bao*.

[7] Zhao C., Wang X., Cong Y. (2014). Effects of bile acids and the bile acid receptor FXR agonist on the respiratory rhythm in the in vitro brainstem medulla slice of neonatal sprague-dawley rats. *PLoS ONE*.

[8] Lopez-Rodriguez E., Pérez-Gil J. (2014). Structure-function relationships in pulmonary surfactant membranes: from biophysics to therapy. *Biochimica et Biophysica Acta (BBA)—Biomembranes*.

[9] De Luca D., Minucci A., Tripodi D. (2011). Role of distinct phospholipases A2 and their modulators in meconium aspiration syndrome in human neonates. *Intensive Care Medicine*.

[10] De Luca D., Minucci A., Zecca E. (2009). Bile acids cause secretory phospholipase A2 activity enhancement, revertible by exogenous surfactant administration. *Intensive Care Medicine*.

[11] Zecca E., De Luca D., Marras M., Barbato G., Romagnoli C. (2007). Intrahepatic cholestasis of pregnancy and bile acids induced lung injury in newborn infants. *Current Pediatric Reviews*.

[12] Oelberg D. G., Downey S. A., Flynn M. M. (1990). Bile salt-induced intracellular Ca^++^ accumulation in type II pneumocytes. *Lung*.

[13] Su K.-C., Wu Y.-C., Chen C.-S. (2013). Bile acids increase alveolar epithelial permeability via mitogen-activated protein kinase, cytosolic phospholipase A2, cyclooxygenase-2, prostaglandin E2 and junctional proteins. *Respirology*.

[14] Yu L., Ding Y., Huang T., Huang X. (2014). Effect of bile acid on fetal lung in rat model of intrahepatic cholestasis of pregnancy. *International Journal of Endocrinology*.

[15] Zhangxue H., Min G., Jinning Z. (2012). Glycochenodeoxycholate induces rat alveolar epithelial type II cell death and inhibits surfactant secretion in vitro. *Free Radical Biology & Medicine*.

[16] Dobbs L. G., Gonzalez R., Williams M. C. (1986). An improved method for isolating type II cells in high yield and purity. *American Review of Respiratory Disease*.

[17] Gonzalez R. F., Dobbs L. G. (2013). Isolation and culture of alveolar epithelial Type I and Type II cells from rat lungs. *Methods in Molecular Biology*.

[18] Mason R. J., Walker S. R., Shields B. A., Henson J. E., Williams M. C. (1985). Identification of rat alveolar type II epithelial cells with a tannic acid and polychrome stain. *American Review of Respiratory Disease*.

[19] Kuipers F., Claudel T., Sturm E., Staels B. (2004). The Farnesoid X Receptor (FXR) as modulator of bile acid metabolism. *Reviews in Endocrine and Metabolic Disorders*.

[20] Heikkinen J., Mäentausta O., Tuimala R., Ylöstalo P., Jänne O. (1980). Amniotic fluid bile acids in normal and pathologic pregnancy. *Obstetrics & Gynecology*.

[21] Dargaville P. A., Mills J. F. (2005). Surfactant therapy for meconium aspiration syndrome: current status. *Drugs*.

[22] Brouwers L., Koster M. P., Page-Christiaens G. C. (2015). Intrahepatic cholestasis of pregnancy: maternal and fetal outcomes associated with elevated bile acid levels. *American Journal of Obstetrics & Gynecology*.

[23] Sweet D. G., Carnielli V., Greisen G. (2013). European consensus guidelines on the management of neonatal respiratory distress syndrome in preterm infants-2013 update. *Neonatology*.

